# Supplementary material for: Kinome state is predictive of cell viability in pancreatic cancer tumor and cancer-associated fibroblast cell lines
Source: PeerJ. 2024 Aug 28;12:e17797. doi: 10.7717/peerj.17797 (PMC11365483; doi:10.7717/peerj.17797)
Supplement: File S1 [file peerj-12-17797-s001.pdf]

Cell Viability Regression Modeling

In addition to developing classification models, we also generated regression models to try to predict cell response. We tried three types of regression models: GLMnet (Friedman et al., 2010), random forest (Wright and Ziegler, 2017) and XGBoost (Chen and Guestrin, 2016). The hyperparameters and associated sampled ranges were as follows:

- GLMnet:
  - Amount of regularization: [-10 - 0] with log10 transformation
  - Proportion of lasso penalty: [0 - 1]
- Random Forest:
  - Number of trees: [1000 - 5000]
  - Minimum data points for split: [2 - 40]
  - Number of predictors sampled: 1 or 2
- XGBoost:
  - Number of trees: [1 - 2000]
  - Maximum tree depth: [1 - 15]
  - Minimum data points for split: [2 - 40]
  - Reduction in loss for required for split: [-10 - 1.5] with log10 transformation
  - Portion of data in fitting: [0.1 - 1]
  - Number of predictors sampled: 1 or 2
  - Learning rate: [-10 - -1] on log10 scale

By RMSE, the regression models we unable to predict cell response to drug (Supp. Figure 1). This was most true for the low cell viability values in the ∼25%-75% range where none of the regression methods were able to accurately predict which compound/concentration combination would result in low cell viabilities. Each of the optimized model predictions did show predictions that on average decreased for low actual cell viabilities (each green line slope is positive in Supp. Figure 1), but this reassuring result was not strong enough to reliably identify new compounds predicted to have strong effects on cell viability.
