# Supplementary material for: Kinome state is predictive of cell viability in pancreatic cancer tumor and cancer-associated fibroblast cell lines
Source: PeerJ. 2024 Aug 28;12:e17797. doi: 10.7717/peerj.17797 (PMC11365483; doi:10.7717/peerj.17797)
Supplement: Figure S1 — The results of the RMSE optimized regression models shown as hex binned heatmaps. Each column shows the optimized results for each cell line, while the rows show the type of model. The dot-dash lines show where a perfect set of predictions would appear, while the green lines show the linear best fit through the presented data points. The RMSE value is also presented in the lower corner of each plot. [file peerj-12-17797-s002.pdf]

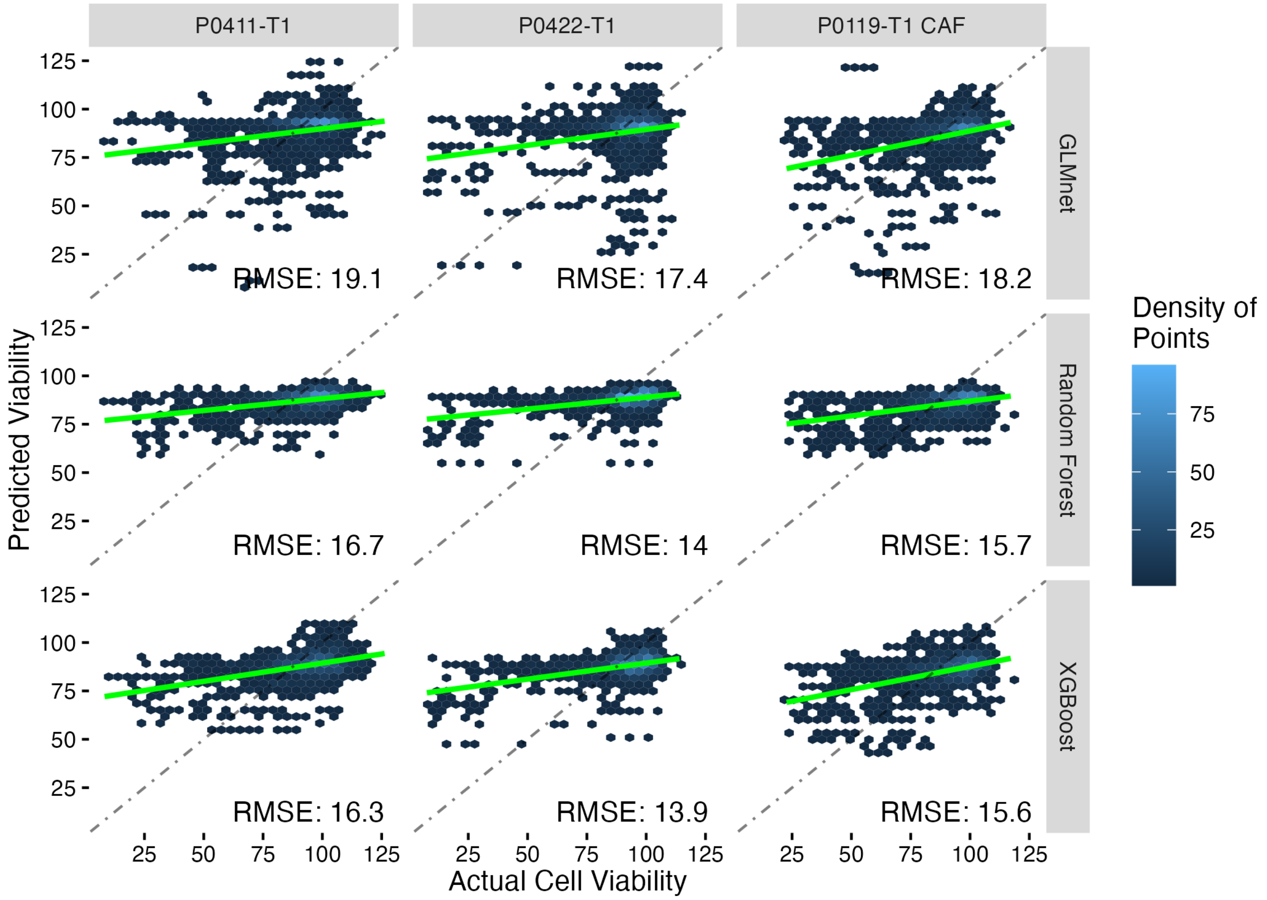

Supplemental Figure 1. Regression models were ineffective at predicting cell viability. The results of the RMSE optimized regression models shown as hex binned heatmaps. Each column shows the optimized results for each cell line, while the rows show the type of model. The dot-dash lines show where a perfect set of predictions would appear, while the green lines show the linear best fit through the presented data points. The RMSE value is also presented in the lower corner of each plot.
